# The Apolipoprotein E ε4 Allele-Dependent Relationship Between Serum Lipid Levels and Cognitive Function: A Population-Based Cross-sectional Study

**DOI:** 10.3389/fnagi.2020.00044

**Published:** 2020-03-13

**Authors:** Shan Wei, Ling Gao, Yu Jiang, Suhang Shang, Chen Chen, Liangjun Dang, Jin Wang, Kang Huo, Jingyi Wang, Qiumin Qu

**Affiliations:** ^1^Department of Neurology, The First Affiliated Hospital of Xi’an Jiaotong University, Xi’an, China; ^2^Department of Neurology, Huxian Hospital of Traditional Chinese Medicine, Xi’an, China

**Keywords:** Alzheimer’s disease, total cholesterol, high-density lipoprotein, low-density lipoprotein, triglyceride, apolipoprotein E, risk factors

## Abstract

**Objectives**: Till now, the effect of serum lipid levels on cognitive function is still controversial. The apolipoprotein E (APOE) ε4 allele is the most critical genetic risk factor for Alzheimer’s disease (AD) and cognitive impairment. Additionally, *APOE* ε4 allele has a major impact on lipid metabolism. The aim of this study was to investigate the *APOE* genotype-dependent relationship between peripheral serum lipid levels and cognitive impairment.

**Methods**: A total of 1,273 subjects aged 40–86 years participated in this cross-sectional study. Serum lipid levels and the *APOE* genotype were detected. Mini-Mental State Examination was used to diagnose the cognitive impairment or not. Univariate and multivariate analyses were used to analyze the relationships between *APOE* genotype, serum lipid levels, and cognition function.

**Results**: After controlling for all possible covariates, a significant interaction between low serum high-density lipoprotein and the *APOE* ε4 allele on cognitive impairment (Wald’s χ^2^ = 4.269, *df* = 1, OR = 20.094, *p* = 0.039) was found in the total participants. In *APOE* ε4 carriers, low serum high-density lipoprotein was positively associated with cognitive impairment (Wald’s χ^2^ = 8.200, *df* = 1, OR = 60.335, *p* = 0.004) and serum high-density lipoprotein levels were positively correlated with Mini-Mental State Examination score (*r* = 0.217, *df* = 176, *p* = 0.004). There was no significant correlation between serum total cholesterol (TC), low-density lipoprotein, triglycerides (TG) levels, and cognitive impairment in either the total participants or *APOE* ε4 carriers/non-carriers.

**Conclusions**: *APOE* ε4 carriers, but not non-carriers, with lower serum high-density lipoprotein had a higher prevalence of cognitive impairment and a lower Mini-Mental State Examination score. These results suggest that the *APOE* ε4 allele may affect the relationship between serum lipid levels and cognitive impairment. However, the specific mechanism needs to be further elucidated.

## Introduction

Alzheimer’s disease (AD) is the most common type of dementia, and its prevalence has been increasing rapidly in China (Jia et al., 2014). A recent study revealed that, in China, the total annual socioeconomic costs of AD patients were US $167.74 billion in 2015 and were predicted to reach US $1.89 trillion in 2050, imposing an immense burden on patients and their families (Jia et al., 2018). Due to the complexity of the pathogenesis of AD, there are currently no unified and effective approaches for preventing or curing AD.

In the brain, amyloid-β (Aβ) accumulation and the formation of insoluble extracellular senile plaques are pathological hallmarks of AD. Aβ is produced by the endoproteolysis of amyloid precursor protein (APP). When cleaved by β-secretase and γ-secretase, APP is mainly hydrolyzed into the 38–43 amino acid residue Aβ peptide (De Felice and Ferreira, 2002), and senile plaques are mainly composed of amyloid Aβ_40_ and amyloid Aβ_42_ (Masters et al., 1985). Researchers have found that reducing cellular cholesterol levels appears to inhibit β-secretase and γ-secretase activity and, thus, decrease the amount of Aβ secreted by neurons (Wahrle et al., 2002; Subasinghe et al., 2003). The responsiveness of Aβ production to cholesterol levels suggests that cholesterol metabolism plays an essential role in the pathogenesis of AD (Hartmann et al., 2007). However, there are still no consistent results of epidemiological studies concerning the role of cholesterol as a risk factor for AD, although some studies have demonstrated that elevated cholesterol increases the risk of AD development, particularly in middle-aged individuals (Kivipelto et al., 2001; Whitmer et al., 2005). Other researchers have failed to confirm this result (Reitz et al., 2004; Tukiainen et al., 2012).

Apolipoprotein E (*APOE*) ε4 is the most important genetic risk factor for AD. *APOE* is a polymorphic protein involved in the development of late-onset AD, although the mechanism has not been fully elucidated (Siest et al., 2000). There are three alleles of *APOE* (E2, E3, and E4) that produce three homozygous (E2/2, E3/3, and E4/4) and three heterozygous (E2/3, E2/4, and E3/4) isoforms (Zannis and Breslow, 1981). Pathophysiological studies show that *APOE* immunoreactivity exists in senile plaques, indicating a significant role of *APOE* in the metabolism of Aβ (Kim et al., 2009; Castellano et al., 2011), which is thought to initiate toxic events and further have distinct functions in regulating tau hyperphosphorylation, synaptic plasticity, cell signaling, lipid transport and metabolism, and neuroinflammation (Yu et al., 2014; Giau et al., 2015). Additionally, *APOE* ε4 has a major impact on lipid metabolism (Mahley, 1988). The *APOE* gene modulates serum concentrations of lipid and lipoproteins according to its high affinity for binding to cell-surface lipoprotein receptors. Previous research has reported that carriers of *APOE* ε*4* have a higher risk of hyperlipidemia (Dallongeville et al., 1992).

Considering that the *APOE* genotype plays an important role in both AD pathogenesis and lipid metabolism, we investigated the effects of the *APOE* genotype on the relationships between peripheral serum lipid levels and cognitive impairment in Chinese middle-aged and elderly subjects from Qubao village in the suburbs of Xi’an, northwest China.

## Materials and Methods

### Participants

**Figure 1:**
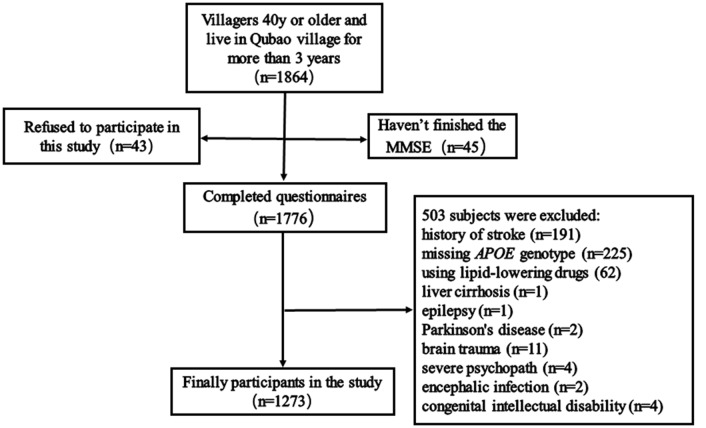
Flow chart of participant selection. Qubao village: the suburbs of Xi’an in northwest China. *APOE*, apolipoprotein E.

Between December 2016 and April 2017, 1,865 subjects were recruited from Qubao village in the suburbs of Xi’an, China. Inclusion criteria were as follows: being 40 years old or older; having lived in Qubao for more than 3 years; agreeing to participate in the study and completing the questionnaire; and having venous blood collected. The exclusion criteria were as follows: (1) participants who had used lipid-lowering drugs in the last 3 months; (2) participants who had severe liver, kidney, thyroid, and hematopoietic system diseases; (3) participants who had suffered from a clear history of acute cerebrovascular disease, including stroke; (4) participants who had suffered from severe nervous system diseases that can cause cognitive impairment, including infection, Parkinson’s disease, epilepsy, congenital intellectual disability, and craniocerebral operations; (5) participants who had suffered from other physical and chemical factors that led to cognitive impairment (drug poisoning, alcoholism, and carbon monoxide poisoning); (6) participants who had suffered from severe psychopathy, including schizophrenia, bipolar disorder, severe depression or anxiety; and (7) participants without a complete MMSE score, biomarkers, or covariates. Considering all the inclusion and exclusion criteria, 1,273 subjects were included in our study (Figure 1).

### Questionnaire Survey

All of the participants were asked to complete the questionnaire survey in a structured in-person interview given by trained interviewers. The questionnaire included basic information (gender, age, education, and marital status), lifestyle (exercise, smoking, and alcohol consumption), and medical history (hypertension, diabetes, dyslipidemia, cerebrovascular disease, and heart disease). The definitions of hypertension and diabetes are consistent with those in our previous articles (Jia et al., 2018). According to the diagnostic criteria of Chinese adult dyslipidemia prevention guide (2007 edition; Joint Committee for Developing Chinese guidelines on Prevention and Treatment of Dyslipidemia in Adults, 2007), people who meet any of the following criteria can be defined as dyslipidemia: high total cholesterol (TC): TC ≥ 5.18 mmol/L; high low-density lipoprotein (LDL-c): LDL-c ≥ 3.37 mmol/L; low high-density lipoprotein (HDL-c): HDL-*c* < 1.04 mmol/L; high triglycerides (TG): TG ≥ 1.70 mmol/L. In addition, anthropometric measurements of participants were taken at the scene, including blood pressure, pulse rate, height, weight, and waist and hip circumference. The body mass index (BMI) was weight (kg) divided by the square of height in meters, and the waist–hip ratio was the waist circumference divided by the hip circumference.

### Diagnosis of Cognitive Impairment

The Mini-Mental State Examination (MMSE) questionnaire was completed by the participants to assess global cognitive function. The MMSE was chosen by Katzman et al. (1988) based on a Chinese version that had been shown to have good sensitivity and specificity. Participants with scores below the cutoff value were considered to have cognitive impairment. The cutoff values were as follows: scores ≤17 for illiteracy, scores ≤20 for participants who only finished primary school, and scores ≤24 for participants with junior high school or above level of education.

### Biochemical Assessment and *APOE* Genotype Detection

After completing the questionnaire, 10 ml of blood was extracted from the elbow vein of each participant under the condition of fasting for more than 8 h, which was put into a purple-top EDTA-anticoagulant tube and a red-top non-anticoagulant tube. The red-top tube blood samples were sent to the biochemical laboratory of the First Affiliated Hospital of Xi’an Jiaotong University for biochemical assessment [HDL-c, LDL-c, TG, TC, and fasting blood glucose (FBG)]. The concentrations of serum HDL-c, LDL-c, TC, TG, and FBG levels were tested by enzymatic method using an automated biochemical analyzer (C501, Roche, Sweden). The purple-top tube blood samples were centrifuged within 2 h after collection at a rate of 3,000 revolutions per second for 10 min, and all of the samples were stored in the refrigerator at −80°C for future analysis. An extraction kit (Tiangen Co. Beijing, China) was used to extract DNA from the frozen EDTA–anticoagulant blood according to the manufacturer’s protocol. Using human genome DNA as a template, the 244-bp length of the target DNA fragment that included two polymorphic sites at amino acid residues 112 and 158 (Mahley and Rall, 2000) was amplified by a PCR thermocycler. All PCR products were detected by Sanger sequencing (Sangon Company, Shanghai, China) to finally determine the *APOE genotype*.

### Statistical Analyses

SPSS 18.0 software (SPSS Inc., IBM, Chicago, IL, USA) was used to analyze all of the data. First, participants were divided into a dyslipidemia group and a non-dyslipidemia group. In addition, according to the *APOE* genotype, the participants could also be divided into *APOE* ε4 non-carriers (E2/2, E2/3, and E3/3) or *APOE* ε4 carriers (E2/4, E3/4, and E4/4). Unpaired Student’s *t*-tests and the mean ± SD were used for data that were approximately normally distributed; the Mann–Whitney *U* test and the median (quartiles) were used for skewed data distributions, and the Pearson χ^2^ test and percentages were used for categorical data. A *p*-value of <0.05 (two-tailed) was considered statistically significant.

Then, a χ^2^ test or Fisher’s exact test was used to compare the differences in cognitive impairment between serum lipid groups in the total participants and in the subgroups according to *APOE* ε4 status. For multivariate analysis, binary logistic regression was used to correct for covariates, including gender, age, education years, smoking, drinking, physical activity, medical history, FBG, mean arterial pressure (MAP), pulse rate, and BMI.

Finally, partial correlation analysis was used to research the correlations between MMSE score and serum lipid levels in the subgroups according to the *APOE* ε4 status. The covariates included age, sex, education years, smoking, drinking, intensity of physical activity, BMI, log-transformed FBG, MAP, pulse rates, and heart disease.

## Results

### Demographic Characteristics of the Study Samples

A total of 1,273 subjects ranging from 40 to 86 years old (mean 57.1 ± 9.7 years) were included in the study, including 755 (59.3%) women. Participants with dyslipidemia comprised 59.2% (754) of the total population. Ninety-nine (7.8%) subjects met the diagnostic criteria of cognitive impairment, and a total of 189 people (14.8%) were *APOE* ε4 carriers. Table 1 shows the demographic characteristics of the total participants. There were significant differences in gender, education, smoking, hypertension, diabetes mellitus status, MAP, pulse rate, FBG level, BMI, and *APOE* ε4 carrier status between the dyslipidemia group and the normal lipids group.

**Table 1:** Demographic and clinical characteristics of the study samples.

	Total (*n* = 1,273)	Normal serum lipids (*n* = 519)	Dyslipidemia (*n* = 754)	*df*	*p*-value
Age, years (mean ± SD)	57.1 ± 9.7	56.5 ± 10.2	57.5 ± 9.3	1,271	0.062
Gender, female (%)	755 (59.3)	275 (53.0)	480 (63.7)	1	<0.001
Education, years (mean ± SD)	6.4 ± 3.4	6.8 ± 3.2	6.2 ± 3.5	1,271	0.003
Drinking, *n* (%)	153 (12.0)	68 (13.1)	85 (11.3)	1	0.324
Smoking, *n* (%)	400 (31.4)	187 (36.0)	213 (28.2)	1	0.003
Lack of activity, *n* (%)	252 (19.8)	97 (18.7)	155 (12.2)	1	0.411
Cardiovascular disease, *n* (%)	114 (9.0)	47 (9.1)	67 (8.9)	1	0.917
Hypertension, *n* (%)	479 (37.6)	164 (31.6)	315 (41.8)	1	<0.001
Diabetes mellitus, *n* (%)	142 (11.2)	51 (9.8)	91 (12.1)	1	0.212
MAP, mmHg (mean ± SD)	95.7 ± 11.7	94 ± 12	97 ± 12	1,271	<0.001
Pulse rate, (mean ± SD)	74 ± 10	73 ± 10	75 ± 10	1,271	<0.001
FBG, mmol/L, median (quartile)	5.34 (5.02, 5.80)	5.27 (4.98, 5.66)	5.40 (5.05, 5.89)	—	<0.001
BMI, kg/m^2^ (mean ± SD)	25.10 ± 3.20	24.32 ± 3.06	25.62 ± 3.19	1,271	<0.001
Cognitive impairment, *n* (%)	99 (7.8)	41 (7.9)	58 (7.7)	1	0.892
*APOE* ε4 carriers, *n* (%)	189 (14.8)	57 (11.0)	132 (10.4)	1	0.001

*Unpaired Student’s t-test and mean ± SD were used to compare the difference of the approximately normally distributed continuous variables between normal serum lipids group and dyslipidemia group. Mann–Whitney U test and median (quartile) were used for the skew distributional data, and *χ*^2^ and percentage were used for categorical variables. SD, standard deviation; MAP, mean arterial pressure; FBG, fasting blood glucose; BMI, body mass index; *APOE*, apolipoprotein E*.

### The Current Prevalence of Cognitive Impairment Between the Normal Serum Lipids Group and the Dyslipidemia Group

**Figure 2:**
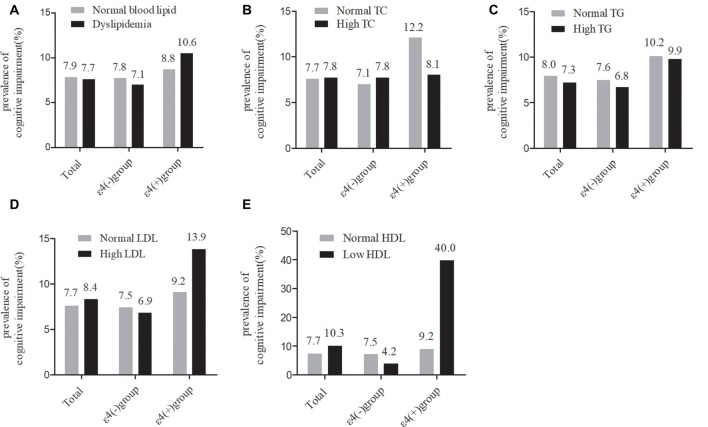
Comparison of the differences in cognitive impairment between serum lipid groups in the total participants and in subgroups according ApoEε4 status. TC, total cholesterol; LDL-c, low-density lipoprotein; HDL-c, high-density lipoprotein; TG, triglycerides; *APOE*, apolipoprotein E.** (A–D)** χ^2^ test. **(E)** Fisher’s exact test.

As shown in Figure 2, in the total samples, the current prevalence of cognitive impairment was not significantly different between the normal serum lipids group and the dyslipidemia group. The stratified analyses according to *APOE* ε4 status showed that the current prevalence of cognitive impairment was still not significantly different between any of the serum lipid groups in *APOE* ε4 carriers or non-carriers.

### Multivariate Analysis of the Relationship Between Serum Lipid Parameters and Cognitive Impairment in the Total Samples

To eliminate the influence of covariates, binary logistic regression analysis was performed. In the total samples, after adjusting for age, gender, and education, no significant correlation was found between high TG, high TC, high LDL, low HDL levels, and cognitive impairment (Table 2, Model 1). When continuing to bring other covariates into the binary logistic regression models, the results were approximately the same (Table 2, Model 2). However, with the binary logistic regression analysis of the interaction between the serum lipids and *APOE* genotype on cognitive impairment, we found that the interaction between low HDL and *APOE* ε4 status was positively correlated with cognitive impairment (Wald’s χ^2^ = 4.269, *df* = 1, OR = 20.094, 95% CI = 1.167–346.056, *p* = 0.039), and the interaction between other serum lipid parameters (high TG, high TC, and high LDL levels) and *APOE* ε4 status had no significant effect on cognitive impairment (Table 2, Model 3).

**Table 2:** The relationships between serum lipid parameters and cognitive impairment with binary logistic regression in the total samples.

		*B*	SE	Wald’s *χ*^2^	OR	95% CI	*p*-value
Model 1							
	High TC	−0.195	0.222	0.771	0.823	0.533–1.271	0.380
	High TG	−0.167	0.239	0.487	0.846	0.529–1.353	0.485
	High LDL-c	−0.062	0.312	0.039	0.940	0.511–1.732	0.843
	Low HDL-c	0.595	0.655	0.824	1.813	0.502–6.544	0.364
Model 2							
	High TC	−0.161	0.226	0.506	0.851	0.546–1.326	0.477
	High TG	−0.144	0.250	0.333	0.866	0.531–1.413	0.564
	High LDL-c	−0.061	0.319	0.036	0.941	0.504–1.757	0.849
	Low HDL-c	0.718	0.655	1.203	2.051	0.568–7.404	0.273
Model 3							
	High TC	−0.077	0.248	0.096	0.926	0.569–1.507	0.757
	High TC by *APOE* ε4 status	−0.462	0.568	0.662	0.630	0.207–1.918	0.416
	High TG	−0.174	0.279	0.390	0.840	0.486–1.452	0.532
	High TG by *APOE* ε4 status	0.146	0.592	0.061	1.157	0.363–3.692	0.805
	High LDL-c	−0.180	0.382	0.222	0.835	0.395–1.766	0.637
	High LDL-c by *APOE* ε4 status	0.435	0.704	0.381	1.544	0.389–6.136	0.537
	Low HDL-c	−0.354	1.061	0.111	0.702	0.088–5.614	0.739
	Low HDL-c by *APOE* ε4 status	3.000	1.452	4.269	20.094	1.167–346.056	0.039

*Binary logistic regression model was used for data analysis, df = 1. APOE ε4 carrier status: dummy coded with ε4 carriers = 1, non-carriers = 0. Model 1 was adjusted for age, gender, and education years. Model 2 was adjusted for age, gender, education years, smoking, drinking, intensity of physical activity, body mass index, log-transformed fasting blood glucose, mean arterial pressure, pulse rate, heart disease, and *APOE* ε4 carrier status. Model 3 was adjusted for the covariates included in model 2 as well as the interaction terms of *APOE* ε4 carrier status by serum lipids. TC, total cholesterol; LDL-c, low-density lipoprotein; HDL-c, high-density lipoprotein; TG, triglycerides; *APOE*, apolipoprotein E*.

### The Effects of the *APOE* ε4 Allele on Cognitive Impairment and Serum Lipid Levels

Univariate analysis showed that compared with *APOE* ε4 non-carriers, serum TC, TG, and LDL levels were higher and HDL levels were lower in *APOE* ε4 carriers [TC: 5.36 ± 0.94 mmol/L vs. 5.15 ± 0.99 mmol/L, *df* = 1,271, *p* = 0.008; TG: 1.48 (1.09, 2.15) mmol/L vs. 1.29 (0.96, 1.80) mmol/L, *p* < 0.001; LDL: 2.82 ± 0.62 mmol/L vs. 2.61 ± 0.66 mmol/L, *df* = 1,271, *p* < 0.001; HDL: 1.53 ± 0.32 mmol/L vs. 1.60 ± 0.34 mmol/L, *df* = 1,271, *p* = 0.007], and the prevalence of dyslipidemia was also significantly higher in *APOE* ε4 carriers [132 (69.8%) vs. 622 (57.4%), *df* = 1, *p* = 0.001]. The current prevalence of cognitive impairment was not significantly different between *APOE* ε4 carriers and non-carriers. Other covariates (age, gender, degree of education, smoking, drinking, intensity of physical activity, hypertension, diabetes mellitus, coronary heart disease, MAP, and BMI) also showed no significant difference between the two groups (Table 3).

**Table 3:** Difference of the cognitive impairment, serum lipid levels, and other covariates between *APOE* ε4 carriers and non-carriers.

	*APOE* ε4 non-carriers (*n* = 1,084)	*APOE* ε4 carriers (*n* = 189)	*df*	*p*-value
Age, years (mean ± SD)	57.0 ± 9.8	57.5 ± 9.3	1,271	0.501
Gender, female (%)	642 (59.2)	113 (59.8)	1	0.884
Education, years (mean ± SD)	6.5 ± 3.4	6.2 ± 3.5	1,271	0.228
Drinking, *n* (%)	133 (12.3)	20 (10.6)	1	0.510
Smoking, *n* (%)	343 (31.6)	57 (30.2)	1	0.685
Lack of activity, *n* (%)	217 (20.0)	35 (18.5)	1	0.633
Cardiovascular disease, *n* (%)	103 (9.5)	11 (5.8)	1	0.102
Hypertension, *n* (%)	410 (37.8)	69 (36.5)	1	0.731
Diabetes mellitus, *n* (%)	116 (10.7)	26 (13.8)	1	0.218
MAP, mmHg (mean ± SD)	95.7 ± 11.8	95.6 ± 11.3	1,271	0.860
Pulse rate (mean ± SD)	74 ± 10	74 ± 9	1,271	0.771
FBG, mmol/L, median (quartile)	5.33 (5.01, 5.80)	5.36 (5.04, 5.88)	—	0.543
BMI, kg/m^2^ (mean ± SD)	25.07 ± 3.15	25.26 ± 3.47	1,271	0.437
Cognitive impairment, *n* (%)	80 (7.4)	19 (10.1)	1	0.205
Dyslipidemia, *n* (%)	622 (57.4)	132 (69.8)	1	0.001
TC, mmol/L (mean ± SD)	5.15 ± 0.99	5.36 ± 0.94	1,271	0.008
TG, mmol/L, median (quartile)	1.29 (0.96, 1.80)	1.48 (1.09, 2.15)	—	<0.001
LDL-c, mmol/L (mean ± SD)	2.61 ± 0.66	2.82 ± 0.62	1,271	<0.001
HDL-c, mmol/L (mean ± SD)	1.60 ± 0.34	1.53 ± 0.32	1,271	0.007

*Unpaired Student’s *t*-test and mean ± SD were used to compare the difference of the approximately normally distributed continuous variables between *APOE* ε4 carriers and non-carriers. Mann-Whitney *U* test and median (quartile) were used for the skew distributional data. *χ*^2^ and percentage were used for categorical variables. SD, standard deviation; MAP, mean arterial pressure; FBG, fasting blood glucose; BMI, body mass index; TC, total cholesterol; LDL-c, low-density lipoprotein; HDL-c, high-density lipoprotein; TG, triglycerides; *APOE*, apolipoprotein E*.

### Stratified Multivariate Analysis of the Relationship Between Serum Lipids and Cognitive Impairment According to *APOE* ε4 Status

Because the *APOE* ε4 allele had effects on the relationship between serum lipid levels and cognitive impairment, stratified binary logistic regression analyses were performed according to *APOE* ε4 status. In *APOE* ε4 carriers, low HDL was positively correlated with cognitive impairment (Wald’s χ^2^ = 8.200, *df* = 1, OR = 60.335, 95% CI = 3.646–998.364, *p* = 0.004; Table 4, Model 5). However, such a correlation disappeared among *APOE* ε4 non-carriers (Wald’s χ^2^ = 0.057, *df* = 1, OR = 0.776, 95% CI = 0.097–6.221, *p* = 0.811; Table 4, Model 7). For other serum lipid parameters (high TC, high TG, and high LDL levels), there was no significant correlation with cognitive impairment in either *APOE* ε4 carriers or non-carriers (Table 3, Models 5 and 7).

**Table 4:** The relationships between serum lipid parameters and cognitive impairment with binary logistic regression in the subgroups according *APOE*ε4 status.

Participants		*B*	SE	Wald’s *χ*^2^	OR	95% CI	*p*-value
*APOE* ε4 carriers	Model 4						
	High TC	−0.716	0.545	1.724	0.489	0.168–1.423	0.189
	High TG	0.098	0.556	0.031	1.103	0.371–3.284	0.860
	High LDL-c	0.166	0.618	0.072	1.181	0.352–3.965	0.788
	Low HDL-c	3.172	1.203	6.957	23.847	2.259–251.772	0.008
	Model 5						
	High TC	−0.946	0.594	2.540	0.388	0.121–1.243	0.111
	High TG	−0.238	0.615	0.150	0.788	0.236–2.628	0.698
	High LDL-c	0.060	0.665	0.008	1.062	0.288–3.914	0.928
	Low HDL-c	4.100	1.432	8.200	60.335	3.646–998.364	0.004
*APOE* ε4 non-carriers	Model 6						
	High TC	−0.103	0.245	0.176	0.902	0.558–1.458	0.675
	High TG	−0.247	0.270	0.840	0.781	0.460–1.325	0.359
	High LDL-c	−0.219	0.376	0.339	0.803	0.384–1.680	0.561
	Low HDL-c	−0.453	1.057	0.184	0.636	0.080–5.043	0.668
	Model 7						
	High TC	−0.052	0.250	0.043	0.950	0.582–1.549	0.836
	High TG	−0.176	0.283	0.385	0.839	0.482–1.461	0.535
	High LDL-c	−0.166	0.383	0.187	0.847	0.400–1.795	0.665
	Low HDL-c	−0.254	1.062	0.057	0.776	0.097–6.221	0.811

*Binary logistic regression model was used for data analysis, df = 1. Models 4 and 5 were analyzed in *APOE* ε4 carriers. Models 6 and 7 were analyzed in *APOE* ε4 non-carriers. *APOE* ε4 carrier status: dummy coded with ε4 carriers = 1, non-carriers = 0. Models 4 and 6 were adjusted for age, gender, and education years. Models 5 and 7 were adjusted for age, gender, education years, smoking, drinking, intensity of physical activity, body mass index, log-transformed fasting blood glucose, mean arterial pressure, pulse rate, and heart disease. TC, total cholesterol; LDL-c, low-density lipoprotein; HDL-c, high-density lipoprotein; TG, triglycerides; *APOE*, apolipoprotein E*.

### Stratified Multivariate Analysis of the Correlations Between Serum Lipids and MMSE Score According to *APOE* ε4 Status

**Figure 3:**
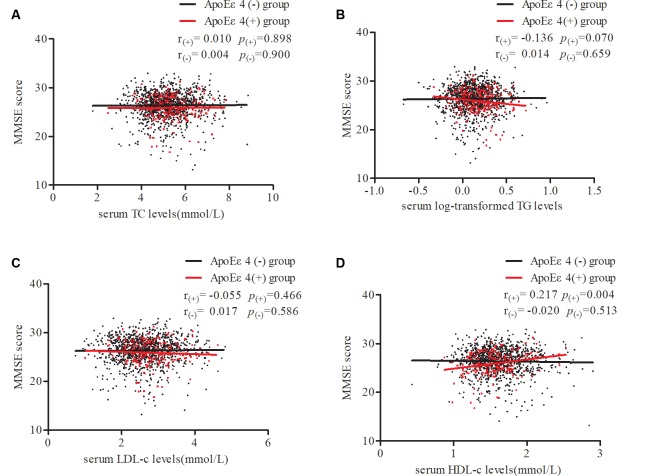
Partial correlations of MMSE score and serum lipid levels according *APOE* ε4 status. Participants were divided into two groups: *APOE* ε4 carriers and non-carriers, which are represented by red dots and black dots. Partial linear correlations between MMSE scores and serum TC **(A)**, log-transformed TG **(B)**, LDL-c **(C)**, and HDL-c **(D)** levels are shown in the figure. r_(+)_ and *p*_(+)_ represent partial correlation coefficients in carriers, while r_(-)_ and *p*_(-)_ represent non-carriers. Adjusted for age, sex, education, smoking, drinking, intensity of physical activity, body mass index (BMI), log-transformed fasting blood glucose (FBG), mean arterial pressure, pulse rates, and heart disease. TC, total cholesterol; TG, triglycerides; LDL-c, low-density lipoprotein; HDL-c, high-density lipoprotein; *APOE*, apolipoprotein E.

To further confirm our results, stratified partial correlation analysis was separately performed to research the correlations between serum lipids and MMSE score in *APOE* ε4 non-carriers and carriers. When adjusting for age, sex, education, smoking, drinking, intensity of physical activity, BMI, log-transformed FBG, MAP, pulse rates, and heart disease, MMSE scores positively correlated with serum HDL level (*r* = 0.217, *df* = 176, *p* = 0.004) but not with LDL, TG, or TC levels in *APOE* ε4 carriers. In *APOE* ε4 non-carriers, no correlations were found between MMSE scores and any of the serum lipids (Figure 3).

## Discussion

In this cross-sectional study, we found a significant interaction effect between low HDL and the *APOE* ε4 allele on cognitive impairment in the total participants. In *APOE* ε4 carriers, low serum HDL-c levels were positively associated with cognitive impairment, and participants with higher HDL levels had higher MMSE scores. No correlation was found between cognitive impairment/MMSE score and any of the serum lipids in *APOE* ε4 non-carriers.

Although studies have shown that lipid metabolism is involved in the pathogenesis of AD (Hartmann et al., 2007; Anstey et al., 2008), and the effect of lipid levels on cognitive function has attracted the attention of many researchers, there is still no consistent conclusion. A previous study showed that high midlife TC levels can increase the risk of AD in older people (Anstey et al., 2017). In contrast, another study showed that higher TC levels were associated with better memory functioning in very elderly subjects without the *APOE* ε4 allele (West et al., 2008). One study showed that increased serum LDL levels were independently associated with AD (Chen et al., 2019), and one study showed that higher LDL levels were associated with better memory performance (Leritz et al., 2016). Katsumata et al. (2013) reported that HDL levels and lower TG/HDL-C ratios were associated with better memory performance, while other studies have shown no association between HDL and AD (Li et al., 2005). Therefore, to study the relationship between lipids and cognition, stratified analysis was separately performed according to the *APOE* genotype. Our study found that low HDL was associated with cognitive impairment in *APOE* ε4 allele carriers, while TC, LDL, and TG levels were not associated with cognitive function.

Some possible mechanisms might explain the positive correlation between HDL levels and cognitive function. First, it has been shown that ApoA-I, the main component of HDL, is highly efficient at cholesterol efflux in the CSF (Demeester et al., 2000), resulting in increased membrane fluidity that could enhance the α-secretase cleavage of APP at the cell membrane. As mentioned above, increased α-secretase activity can reduce the production of Aβ. Second, apoA-I/HDL can bind to Aβ and make the clearance of Aβ by astrocytes and/or microglia more efficient (Sagare et al., 2012), leading to a decrease in Aβ aggregation and cytotoxicity. Third, it is well established that the antioxidant and anti-inflammatory effects of HDL play an important role in cardiovascular diseases (Barter et al., 2007), so the same disease mechanism may also affect neurodegenerative diseases (Lewis et al., 2010). Finally, the main role of HDL is lipid metabolism, which can reverse lipoprotein transport from the arterial wall of the brain (Mulder and Terwel, 1998), leading to a decrease in ischemic lesions that are involved in the development of cognitive decline and dementia (Kalaria, 2000).

However, the difference in the relationship between HDL and cognitive function between *APOE* ε4 carriers and *APOE* ε4 non-carriers remains to be addressed. We hypothesized that in *APOE* ε4 carriers, there may be two reasons for the positive association between cognitive function and HDL levels. First, this study showed lower levels of HDL in *APOE* ε4 carriers, suggesting that *APOE* exerts isoform-specific effects on HDL metabolism in humans. Using C57BL/6 *APOE*^−/−^ mice, Hopkins et al. (2002) reported that compared with *APOE* ε3, the presence of *APOE* ε4 was less efficient at transferring ApoA-I from chylomicron remnants to HDL, resulting in lower plasma HDL levels and smaller HDL volumes. The reduced HDL levels may be associated with eventual cognitive decline, which has been confirmed by some previous studies (van Exel et al., 2002; Zuliani et al., 2010). However, pathophysiological studies have shown that *APOE* ε4 can directly lead to cognitive decline without HDL involvement by affecting Aβ clearance, tau hyperphosphorylation, synaptic plasticity, cell signaling, and neuroinflammation (Yu et al., 2014; Giau et al., 2015). The combination of these two mechanisms may lead to the emergence of a positive relationship between HDL levels and cognitive function in *APOE* ε4 carriers. However, in *APOE* ε4 non-carriers, without *APOE* ε4 as a risk factor, the effect of HDL alone on cognitive function might not be sufficiently apparent, leading to the result that there was no significant relationship between HDL and cognitive function. Of course, the abovementioned ideas are only our reasonable speculation, and the specific mechanism remains to be further studied.

Consistent with our findings, one community-based study with 2,356 participants showed no correlation between TC levels and the risk of dementia or AD (Elias et al., 2005). In contrast, the 3C study (Schilling et al., 2017) and another 14-year cohort study (Toro et al., 2014) found a positive correlation, and a meta-analysis (Anstey et al., 2017) also showed that high midlife TC levels increase the risk of late-life AD. However, the lipid measurements in these studies were not from cross-sectional studies and were performed significantly earlier than the onset of AD by at least 13 years, and some of the cholesterol levels were measured in midlife and not late life. A previous study of 444 men from the Finnish cohorts of the Seven Countries (Notkola et al., 1998) showed that the TC levels of men in midlife who subsequently developed AD were significantly higher than those of normal men. However, because cholesterol levels decline with age, in the preclinical manifestations of AD stage, the TC levels of those eventually developing AD decreased more rapidly and were eventually lower in late life than those of normal men. This study was a cross-sectional study in Chinese middle-aged and elderly subjects, and all lipid measurements were clustered around the time of the study, which may explain the lack of correlation between TC levels and cognitive impairment.

Some limitations should be mentioned. First, the study is a cross-sectional study, so it can only explain the correlation between blood lipids and cognition; however, the causal relationship between them is difficult to explain, and randomized controlled trial is necessary in the future to see if increasing HDL-c plasma levels can prevent cognitive decline in the at-risk population of *APOE* ε4 carriers and thus clarify the causal relationship. Second, the serum lipids were only detected at a single time point, not allowing for an evaluation of the dynamic changes. In addition, our study focused on elderly individuals in the Chinese Han population in Northwest China, and the diagnosis of cognitive impairment was based on the Chinese version of the MMSE, so the generalizability of our findings may be limited in other ethnic or age groups, and multi-center and large population studies need to be performed to validate our results. Third, with the numbers of patients with cerebrovascular diseases increasing, the incidence rate of vascular dementia (VaD) rises year by year. VaD has become the second leading cause of dementia after AD itself (Lobo et al., 2000). In this study, we have excluded people with a clear history of acute cerebrovascular disease, including stroke. However, owing to lack of standard diagnostic procedures and biomarkers simultaneously (Formichi et al., 2010), it is still hard to rule out the possibility that some cases with cognitive impairment may be vascular dementia or mixed dementia (AD+VaD; Jellinger, 2005). So, further studies involving AD biomarkers need to be performed to elaborate the conclusion more deeply and accurately.

## Conclusion

In summary, by measuring serum lipids and cognitive function, this cross-sectional study found that in *APOE* ε4 carriers, but not *APOE* ε4 non-carriers, low serum HDL-c levels were positively associated with cognitive impairment and that those with higher HDL levels had higher MMSE scores. These data indicated that the *APOE* ε4 allele may affect the relationship between serum lipid levels and cognitive impairment. However, this relationship needs to be further elucidated.

## Data Availability Statement

The raw data supporting the conclusions of this article will be made available by the authors, without undue reservation, to any qualified researcher.

## Ethics Statement

This study and its protocol were approved by the Medical Ethics Committee of the First Affiliated Hospital of Xi’an Jiaotong University. All participants were required to sign a written informed consent form before participating in the study.

## Author Contributions

SW participated in the questionnaire survey and biochemical assessment, conducted the results analysis, and wrote the manuscript. LG and YJ participated in the questionnaire survey, sample collection, and biochemical assessment. SS designed this study and participated in the questionnaire survey and sample collection. CC, LD, JinW, KH, and JingW participated in the questionnaire survey and sample collection. QQ coordinated and supervised all stages of the project. All authors have read and approved the final version of the manuscript.

## Conflict of Interest

The authors declare that the research was conducted in the absence of any commercial or financial relationships that could be construed as a potential conflict of interest.
